# Abnormal T regulatory cells (Tregs: FOXP3^+^, CTLA-4^+^), myeloid-derived suppressor cells (MDSCs: monocytic, granulocytic) and polarised T helper cell profiles (Th1, Th2, Th17) in women with large and locally advanced breast cancers undergoing neoadjuvant chemotherapy (NAC) and surgery: failure of abolition of abnormal treg profile with treatment and correlation of treg levels with pathological response to NAC

**DOI:** 10.1186/1479-5876-11-16

**Published:** 2013-01-15

**Authors:** Chandan Verma, Jennifer M Eremin, Adrian Robins, Andrew J Bennett, Gerard P Cowley, Mohamed A El-Sheemy, Jibril A Jibril, Oleg Eremin

**Affiliations:** 1 Division of Gastrointestinal Surgery, University of Nottingham, E Floor West Block, Queens Medical Centre, Derby Road, Nottingham, NG7 2UH, UK; 2 Research & Development Department, Lincoln Breast Unit, Lincoln County Hospital, Greetwell Road, Lincoln, LN2 5QY, UK; 3 School of Molecular Medical Sciences, A Floor West Block, Queens Medical Centre, Derby Road, Nottingham, NG7 2UH, UK; 4 FRAME Laboratory, School of Biomedical Sciences, University of Nottingham Medical School, Queens Medical Centre, Derby Road, Nottingham, NG7 2UH, UK; 5 Department of Pathology, PathLinks, Lincoln County Hospital, Greetwell Road, Lincoln L, N2 5QY, UK; 6 School of Life Sciences, University of Lincoln, Brayford Pool, Lincoln, LN6 7TS, UK

**Keywords:** Breast cancer, Tregs, Myeloid-derived suppressor cells, Cytokines, Chemotherapy and surgery

## Abstract

**Background:**

Host defences play a key role in tumour growth. Some of the benefits of chemotherapy may occur through modulation of these defences. The aim of this study was to define the status of regulatory cells in women with large and locally advanced breast cancers (LLABCs) undergoing neoadjuvant chemotherapy (NAC) and surgery.

**Methods:**

Bloods were collected from patients (n = 56) before, during and following NAC, and surgery. Controls (n = 10) were healthy, age-matched females donors (HFDs). Blood mononuclear cells (BMCs) were isolated and T regulatory cells (Tregs) (n = 31) determined. Absolute numbers (AbNs) of Tregs and myeloid-derived suppressor cells (MDSCs) were ascertained from whole blood (n = 25). Reverse transcriptase polymerase chain reaction analysis determined Treg mRNA (n = 16). *In vitro* production of Th1, Th2 and Th17 cytokines (n = 30), was documented. Patients were classified as clinical responders by magnetic resonance mammography after two cycles of NAC and as pathological responders using established criteria, following surgery.

**Results:**

Patients with LLABCs had significantly increased circulating Tregs (≥ 6 fold AbN and percentage (%)) and MDSCs (≥ 1.5 fold AbN (p = 0.025)). Percentage of FOXP3^+^ Tregs in blood predicted the response of the LLABCs to subsequent NAC (p = 0.04). Post NAC blood Tregs (%) were significantly reduced in patients where tumours showed a good pathological response to NAC (p = 0.05). Blood MDSCs (granulocytic, monocytic) were significantly reduced in all patients, irrespective of the pathological tumour response to chemotherapy. NAC followed by surgery failed to restore blood Tregs to normal levels. MDSCs, however, were reduced to or below normal levels by NAC alone. *Invitro* Th1 profile (IL-1β, IL-2, INF-γ, TNF-α) was significantly reduced (p ≤ 0.009), whilst Th2 (IL-4, IL-5) was significantly enhanced (P ≤ 0.004). Th1 and Th2 (IL-5) were unaffected by NAC and surgery. IL-17A was significantly increased (p ≤ 0.023) but unaffected by chemotherapy and surgery.

**Conclusion:**

Women with LLABCs have abnormal blood regulatory cell levels (Tregs and MDSCs) and cytokine profiles (Th1, Th2, Th17). NAC followed by surgery failed to abolish the abnormal Treg and Th profiles. There was a significant correlation between the circulatory levels of Tregs and the pathological response of the breast cancers to NAC.

## Background

There is evidence from tumour models in rodents that innate and adaptive immunity play important roles in tumour surveillance, induction and modulation of tumour growth [1-4]. These antitumour host defences may be inadequate, *de novo* or after a period of effective tumour immunoediting, resulting in progressive tumour growth [5,6].

Regulatory T cells (Tregs), macrophages and myeloid-derived suppressor cells (MDSCs) play a crucial role in inhibiting anticancer defences (systemically and in the tumour microenvironment), resulting in tumour escape, progressive growth and metastatic dissemination [7-10].

In man, Tregs (CD4^+^ CD25^+^ FOXP3^+^ (Forkhead Box Protein 3)) have been documented in blood, lymph nodes, ascites and infiltrating the tumour microenvironment in a variety of solid cancers [11-16]. These Tregs inhibit CD4^+^ and CD8^+^ T cells, natural killer (NK, NKT) cells and dendritic cells (DCs) [10,15]. Tregs secrete transforming growth factor-beta (TGF-β) and interleukin-10 (IL-10), which down-regulate antitumour immunity, suppressing the antigen presentation by DCs, CD4^+^ T helper (Th) cell function and the generation of tumour specific CD8^+^ cytotoxic T lymphocytes (CTLs) [10,15]. Through enhanced expression of the cytotoxic T lymphocyte-associated antigen 4 (CTLA-4) in Tregs, the interaction of the CD28 ligand on T lymphocytes with the CD80/86 receptors on DCs is blocked, with downgrading of DC activation and generation of CD8^+^ CTLs, inhibition of IL-12 production and T cell cycle progression [17,18].

MDSCs are a heterogeneous group of leucocytes (monocytic, granulocytic) present in the circulation, lymphoid compartments and infiltrating human cancers [15,19,20]. They secrete TGF-β, IL-10 and inhibit CD4^+^T and CD8^+^T lymphocytes through modulation of L-arginine metabolism and production of reactive oxygen species; they also produce H_2_O_2_ and peroxynitrite. The resultant superoxide radicals damage T cells, reducing their number, inhibiting the T cell receptor complex and inhibiting cell-mediated immune functions [8,15,19,21]. They induce the development of Tregs and T cell anergy [8,22]. They play a crucial role in promoting tumour angiogenesis, tumour invasion and formation of metastases [8].

Many chemotherapeutic agents induce short-lived, inhibitory effects on innate and adaptive immunity [23-25]. However, different drugs and regimens may augment anticancer immunity, both humoral and cellular [23-25].

Chemotherapy-induced cancer cell stress/damage results in the release of immunogenic tumour antigens, as well as ‘danger’ signals (e.g., heat shock proteins), which can activate antigen presenting DCs and other innate cells, respectively, and lead to the release of proinflammatory cytokines, inducing antitumour-specific cell-mediated immune responses [23,25]. Chemotherapeutic agents (e.g., anthracyclines) induce cancer cells to undergo apoptosis with cell surface exposure of calreticulin, thereby, enhancing the capture and uptake of these apoptotic bodies by DCs and subsequent CD8^+^ T cell responses [26].

A number of the side effects (e.g., weakness, myalgia) associated with chemotherapy are due to the systemic release of drug-induced cytokines. Chemotherapeutic agents increased the levels of IL-2, IL-6, interferon-gamma (INF-γ) and decreased the production of IL-1 and tumour necrosis factor-alpha (TNF-α) in women with advanced breast cancer who responded to treatment [27]. Adjuvant treatment of women with breast cancer with paclitaxel, enhanced serum levels of IL-6, IL-8 and IL-10. These enhanced levels were associated with arthralgia and flu-like symptoms [28].

Tregs are reduced substantially by cyclophosphamide (C). Low metronomic doses down-regulate the expression of FOXP3 and glucocorticoid-induced TNF receptor-related proteins and selectively abolish Tregs, without detrimental effects on T effector cells [29,30].

In breast cancer there is evidence of increased circulating levels of Tregs. However, this is poorly defined and there is conflicting data regarding blood levels of Tregs and tumour volume, and the modulatory effect of chemotherapeutic drugs and surgical resection. We have an interest in neoadjuvant chemotherapy (NAC) in women with large and locally advanced breast cancer (LLABC) [31,32]. We have carried out a study evaluating the effect on the pathological response in the breast of the addition of capecitabine (Cap) to docetaxel (T), given sequentially after adriamycin (A) and C. This is an optimum setting to define the status of Tregs (FOXP3^+^, CTLA-4^+^), and MDSCs (monocytic, granulocytic), the cytokine profile (Th1, Th2 and Th17) generated by T cells *in vitro* and the effect of NAC and surgery on these parameters in women with LLABCs.

This study has comprehensively defined the relationship between systemic regulatory cells (Tregs, MDSCs) and LLABC and the prediction of chemoresistance to NAC. The study has also shown convincingly the inability of NAC and surgery to restore the blood Tregs level to that seen in healthy females. These findings suggest the possibility of residual occult metastatic disease and the need for implementing novel therapeutic strategies to restore this persistent dysfunctional immune state and assessing the possible clinical benefits of such therapeutic interventions [30,33,34].

## Methods

### Patients

Fifty six women with LLABCs (>3 cm, T2-T4, N0-2, M0), enrolled in a study of NAC to evaluate the effect of the addition of capecitabine (Cap) to docetaxel (T) preceded by adriamycin and cyclophosphamide (AC), were investigated. After two cycles of AC patients were assessed by magnetic resonance mammography (MRM), compared against pre-NAC MRM, and classified as clinical responders or non-responders. All patients received either 4 courses of AC followed by 4 courses of T ± Cap or 2 courses of AC followed by 6 courses of T ± Cap, as per the trial protocol. All patients underwent surgery (wide local excision or mastectomy and axillary surgery) 4 weeks after the last course of NAC. Pathological responses in the breast and axillary lymph nodes were assessed in the excised surgical specimens after NAC. Pre-NAC assessment was done on core biopsies (breast, lymph nodes), obtained prior to commencement of NAC. An established and previously published grading criteria was used to define histopathological responses in the breast (grades 5 to 1) [32]. Patients were allocated to responder groups accordingly: Group I (grade 5, complete pathological response [cPR]), Group II (grade 4, very good pathological response [VGR]), Group III (grade 3, partial pathological response) and Group IV (grade 2 [very poor] and grade 1 [no] pathological responses). Good pathological responders were Group I and Group II patients, poor pathological responders consisted of Group III and Group IV patients.

Ten healthy age-matched females were used as controls.

The study was given approval by the Leicestershire, Northamptonshire & Rutland Research Ethics Committee 1: Reference Number 07/H0406/260; Favourable Opinion 24/01/2008.

All patients enrolled in the study gave informed consent to participate in and to publish the results of the study. The study Registration is ISRCTN00407556.

### Phenotypic analysis

Blood samples were collected before, during and following completion of NAC, and post-surgery. Blood mononuclear cells (BMCs) were collected on Ficoll-Hypaque, washed and made up in RPMI with 10% foetal calf serum (FCS) (Sigma, UK) and antibiotics (TCM), and stored at -80°C for further analysis (n = 31). Whole blood assays (n = 25) were used for documentation of absolute numbers (AbNs). Controls were 10 age- and sex-matched healthy female donors (HFDs). Flow cytometry (FC) analysis (Beckman Coulter, FC500) was performed with a panel of monoclonal antibodies (MAbs) described below,

FOXP3^+^ Treg BMCs were stained for cell surface markers for 30 minutes with 2.5 ul phycoerythrin Texas red conjugate (ECD)-antihuman CD4, 5 ul phycoerythrin (PE)-antihuman CD25, 5 ul allophycocyanin (APC) antihuman CD127. CTLA-4^+^ Treg BMCs were stained for intracellular CD152. Cell surface markers for CD4 and CD25 were determined by staining for 30 minutes with 2.5 ul of ECD antihuman CD4 and 5 ul fluorescein isothiocyanate (FITC) antihuman CD25. The cells were then washed with RPMI and 2% FCS; 2% formaldehyde was used for fixation of BMCs for 10 minutes at room temperature (RT). The BMCs were then washed once in phosphate buffered saline (PBS) containing 2% FCS, twice in PBS/0.5% Tween with 0.05% azide and 3% FCS. 2.5 ul FITC antihuman FOXP3 (intracellular), and 5 ul PE antihuman CTLA-4 (intracellular CD152) were added to the corresponding tubes and incubated for 2 hours at 4°C (shaking gently every 20 minutes). The BMC pellet was then washed twice in PBS/0.5% Tween, 0.05% azide and 3% FCS. The BMCs were resuspended in 400 μl of 0.5% paraformaldehyde fixative solution for FC analysis.

Whole blood was used to determine absolute numbers of cells. CD4^+^ CD25^+^ Tregs were characterised using 2.5 ul ECD antihuman CD4, 5 ul of PE antihuman CD25. CTLA-4^+^ Tregs were characterised using 2.5 ul ECD antihuman CD4, 5 ul of FITC antihuman CD25 and 5 ul PE antihuman CD152 (intracellular CTLA-4). Monocytic MDSCs were characteristed using 5 ul PE and cyanine dye (PECY-7) antihuman CD11b, 5 ul ECD antihuman CD14, 5 ul ECD antihuman HLA DR, 5 ul PE antihuman CD124 and 5 ul FITC antihuman CD33. Granulocytic MDSCs were characterised using 5 ul PECY-7 anti-human CD11b, 5 ul ECD antihuman CD14, 5 ul PE antihuman CD124, and 5 ul PE antihuman CD15 (Labelled MAbs for Tregs and MDSCc were purchased from Beckman Coulter, UK, Biolegend, UK, and BD Biosciences UK). On adding the MAbs to whole blood a gentle vortex was applied for 5 seconds and the FACs tubes were left in the dark for 15 minutes at RT. 500 ul of optilyse C solution (Beckman Coulter) was added to induce complete lysis of red blood cells, vortexed and left for another 15 minutes at RT in the dark. 500 ul of PBS was added to the FACS tubes to stop the lysis reaction between the optilyse C and the whole blood. The whole blood mixture was vortexed at RT. 100 ul of Flow Count-fluorsphere beads (Beckman Coulter) were added prior to analysis on the flow-cytometer.

CD4^+^ CD25^+^ CD127^-^ cells were purified by cell sorting (MoFlow XDP, Beckman Coulter) by labelling 3 × 10^6^ BMCS/ml with 5 μl PE–antihuman CD25, 2.5 μl PC7-antihuman CD4 and 5 μl APC–antihuman CD127 MAbs. The subset of CD4^+^ CD25^+^ CD127^-^ T cells and CD4^+^CD25^+^CD152^+^ T cells (n = 16) were used for gene expression analysis.

The BD™ CompBeads (BD Biosciences) Anti-Mouse Ig, κ polystyrene microparticles were used to optimize fluorescence compensation settings for multicolor flow cytometric analyses. The set provided two populations of microparticles, the BD™ CompBeads Anti-Mouse Ig, κ particles, which bind any mouse κ light chain-bearing immunoglobulin, and the BD™ CompBeads Negative Control (FCS), which has no binding capacity. When mixed together with a fluorochrome-conjugated mouse antibody, the BD™ CompBeads provide distinct positive and negative (background fluorescence) stained populations which can be used to set compensation levels manually or using instrument set-up software. For each flow cytometric analysis sample an appropriate corresponding tube was set up with a minus fluorescence control (to identify and eliminate all possible non-specific antibody binding and to ensure accurate data acquisition and interpretation was carried out).

### RT-PCR analysis

FOXP3^+^ and CTLA-4^+^ Treg subsets from 16 patients and 8 healthy female donors were used in the analysis. Total RNA was isolated with TRI Reagent, and the first strand cDNA was synthesized using reverse transcriptase (RT) (Invitrogen, UK manufacturer’s instructions). After standard reverse transcription, cDNA was pre-amplified 14 cycles, mRNA expression of FOXP3 and CTLA-4 was determined by quantitative real-time polymerase chain reaction (RT-PCR) – Taq-man RT-PCR, (Applied Biosystems). The Human β-actin gene was the endogenous control for sample normalisation. Results were presented as folds relative to the expression of β-actin.

PCR primary pairs sequences: Human FOXP3: Forward primer (FP) 5-CACCTGGCTGGGA AAATGG-3 and reverse primer (RP) 3-GGAGCCCTTGTCGGATGAT-5; probe sequence (PS) TGACCAAGGCTTCATC; human CTLA-4: FP 5-TGAGTATGCATCTCCAGGCAAA-3; RP3-AGCCTGCCGAAGCACTGT-5; PS CCACTGAGGTCCGGGT; human β-actin: FP-5CCTG GCACCCAGCACAAT-3; RP3GCCGATCCACACGGAGTACT-5; PS ATCAAGATCATTGTCCTCCTGAGCGC.

### *In vitro* cytokine assessments

*In vitro* cytokine assays: CD3^+^ T cells were isolated from BMCs obtained from 30 women with LLABCs and 8 HFDs using micro beads and LS filtration columns placed in the magnetic field of a MACS separator (Miltenyi Biotech). Two × 10^6^ CD3^+^ T cells in TCM were cultured in 24 well plates, at 37°C, with the addition of phorbol 12, 13-dibutyrate (Sigma, UK), 20 ng/ml and ionomycin (Sigma, UK), 0.5μg/ml for 24 hours. Cytokines (IL-1β, IL-2, IL-4, IL-5, IL-6, IL-10, INF-γ, TNF-α) were analysed in the supernatants, using a fluorescent bead immunoassay (Bender Med Systems, Austria). 5 × 10^5^ BMCs were cultured as above and then treated with Brefeldin-A (Sigma, UK) at 10 μg/ml for 24 hours to inhibit secretion of IL-17A. The cells were then washed with RPMI and 2% FCS; 2% formaldehyde was used for fixation of BMCs for 10 minutes at RT. The BMCs were then washed once in PBS containing 2% FCS, twice in PBS/0.5% Tween with 0.05% azide and 3% FCS. A cocktail of antibodies were prepared consisting of 2.5 ul (PeCY7) antihuman CD3, 2.5 ul (ECD) antihuman CD8, 5 ul PE antihuman IL-17A and 2.5 ul FITC antihuman FOXP3. This was added to the corresponding tubes and incubated for 2 hours at 4°C (shaking gently every 20 minutes). The BMC pellet was then washed twice in PBS/0.5% Tween, 0.05% azide and 3% FCS. The BMCs were resuspended in 400 μl of 0.5% paraformaldehyde fixative solution for FC analysis. The % of CD4^+^ IL-17A^+^ ± FOXP3^+^ T cells were determined by FC.

### Statistical analysis

**Figure 1:**
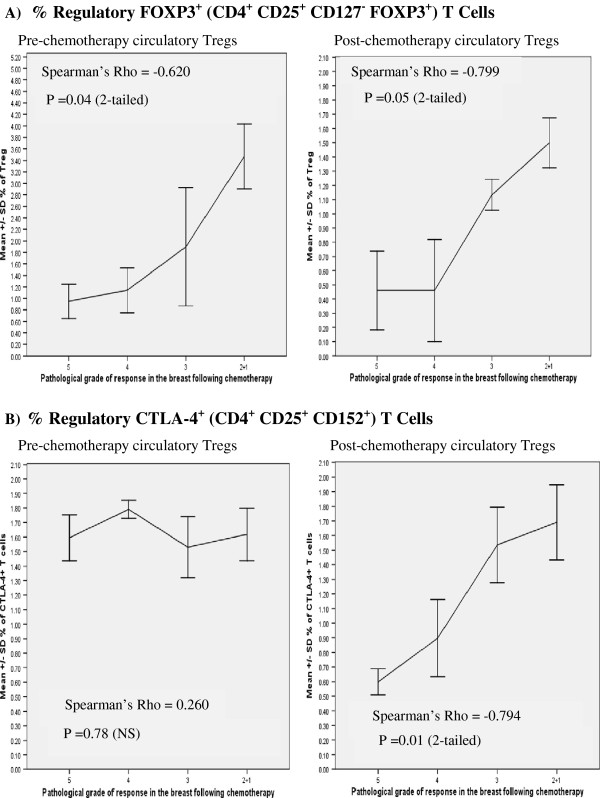
**(A) Correlation between % of regulatory FOXP3**^**+ **^**(CD4**^**+ **^**CD25**^**+ **^**CD127**^**- **^**FOX P3**^**+**^**) T cells before and after NAC and subsequent histological response in the breast following NAC (n = 16), graded as complete (cPR, 5), very good (VG, 4), partial (3), poor and no pathological response (2 + 1) in the breast following NAC.** Values shown are means ± standard deviations. Statistical analysis: Spearman’s correlation coefficient Rho = −0.620, p = 0.04 (2-tailed) (pre-chemotherapy) and Rho = −0.799, p = 0.05 (2-tailed) (post-chemotherapy) **(B)** Correlation between % of regulatory CTLA-4^+^ (CD4^+^ CD25^+^ CD152^+^) T cells before and after NAC and subsequent histological response in the breast following NAC (n = 16), graded as complete (cPR, 5), very good (VG, 4), partial (3), poor and no pathological response (2 + 1) in the breast following NAC. Values shown are means ± standard deviations. Statistical analysis: Spearman’s correlation coefficient Rho = 0.260, p = 0.78 (NS) (pre-chemotherapy) and Rho = −0.794, p = 0.01 (2-tailed) (post-chemotherapy).

Flow cytometry data was analysed using WEASEL version 3.0. All dependent variables were checked by the Shapiro-Wilk Test of Normality to establish the normal distribution or otherwise of the data obtained. SPSS (version 19.0) was used in analysis of data to calculate independent sample t-tests for observing statistical differences. A probability value of equal to or less than 0.05 (p ≤ 0.05) was considered statistically significant. Spearman’s correlation coefficient Rho was used to analyse data (p ≤ 0.05, 2-tailed) in Figure 1 (A & B).

## Results

### Classification of responders

Patients were classified into Groups (I to IV), according to the histopathological responses (see Methods) documented in the breast cancers following NAC, and then categorised into good (Group I and II) and poor (Group III and IV) pathological responders. Patients were also categorised into clinical responder groups (good and poor), depending on the MRM assessment after two courses of AC (see Methods). The latter approach was used in our clinical trial to randomise patients into different treatment groups. It is an early predictor of likely response to NAC.

### Blood Tregs (FOXP3^+^, CTLA-4^+^) in women with LLABCs (n = 56)

The percentage of FOXP3^+^ Tregs (CD4^+^ CD25^+^ CD127^-^ FOXP3^+^) in the blood of 31 women with LLABCs, prior to any treatment, was 1 · 64 ± 0 · 36% (7 fold increase compared with HFDs) (Table 1). The AbN of Tregs (CD4^+^ CD25^+^) in 25 women with LLABCs was 201.33 ± 13.33 cells/μl (6 fold increase compared with HFDs) (Table 1). The lack of the FOXP3 marker in the assay (unavailable when the AbN assays were carried out) is likely to overestimate the AbN of Tregs.

**Table 1:** **Presence of regulatory cells (T cells [FOXP3**^**+**^**, CTLA-4**^**+**^**], MDSCs [Monocytic, Granulocytic]) in women with LLABCs**

**Nature of Regulatory Cells**	% in the Circulation of Women with LLABCs* (n = 31)	% in the Circulation of HFDs** (n = 10)	AbN (cells/μl) in the Circulation of Women with LLABCs* (n = 25)	**AbN (cells/μl) in the Circulation of HFDs (n = 10)**
T regulatory: % (CD4^+^ CD25^+^ CD127^-^ FOXP3^+^); AbN (CD4^+^ CD25^+^)	1.64 ± 0.36*** (7 fold increase)	0.24 ± 0.02	201.33 ± 13.33 (6 fold increase)	35.71 ± 2.71
T regulatory: % (CD4^+^ CD25^+^ CD152^+^); AbN (CD4^+^ CD25^+^ CD152^+^)	1.43 ± 0.08 (7 fold increase)	0.21 ± 0.05	17.00 ± 0.90 (2 fold increase; p = 0.002)	9.75 ± 0.55
MDSCs: AbN monocyte-derived (CD11b^+^ CD14^+^ HLA DR^-^ CD124^+^ CD33^+^)	-	-	28.67 ± 4.33 (3 fold increase)	10.20 ± 2.80
MDSCs: AbN granulocyte-derived (CD11b^+^ CD14^-^ CD66b^+^ CD124^+^ CD15^+^)	-	-	185.13 ± 14.86 (1.5 fold increase; p = 0.025)	129.00 ± 21.00

* LLABCs: Large and locally advanced breast cancers; ** HFDs: Healthy female donors; ***: Values shown for patients with LLABCs and HFDs are Means ± SDs.

The % of CTLA-4^+^ Tregs (CD4^+^ CD25^+^ CD152^+^) in the blood of 31 women with LLABCs was 1.43 ± 0.08% (7 fold increase compared with HFDs) (Table 1). The AbN of CTLA-4^+^ Tregs (CD4^+^ CD25^+^ CD152^+^) in 25 patients was 17.00 ± 0.9 cells/μl (2 fold increase compared with HFDs [p = 0.002]) (Table 1).

### Blood MDSCs (Monocytic, Granulocytic) in women with LLABCs (n = 25)

The AbN of monocytic (CD11b^+^ CD14^+^ HLADR^-^ CD124^+^ CD33^+^) MDSCs in women with LLABCs was 28.67 ± 4.33 cells/μl (3 fold increase compared with HFDs) (Table 1).

The AbN of granulocytic (CD11b^+^ CD14^-^ CD66b^+^ CD124^+^ CD15^+^) MDSCs in 25 women with LLABCs was 192 ± 21.5 (1.5 fold increase compared with HFDs [p = 0.025]) (Table 1).

### Effect of NAC on blood levels of Tregs (FOXP3^+^, CTLA-4^+^) (n = 16)

Four cycles of NAC had no effect on the circulating levels of Tregs (FOXP3^+^, CTLA-4^+^) (data not shown).

After 8 cycles of NAC, there was a significantly reduced % of CD4^+^CD25^+^CD127^-^ FOXP3^+^Tregs, compared with pre-treatment levels, in patients who had a good pathological response in their tumours (Group I and II) (p = 0.033). However, the levels were still abnormally elevated when compared with HFDs, even in those patients whose tumours showed cPRs (p = 0.004). Patients (Groups III and IV) whose tumours underwent a poor pathological response showed no significant change in the circulating levels of FOXP3^+^ Tregs, compared with pre-treatment levels (Table 2).

**Table 2:** Tregs in the blood of women with LLABCs undergoing NAC and subsequent surgery

**Regulatory T Cells**					
**Baseline (B) Levels in LLABCs *Versus *(v) Levels Following Completion of Chemotherapy (CC) in Different Responders**					
**Study Group Comparisons**		**FOXP3**^**+ **^**(%)**	**CD4**^**+ **^**CD25**^**+ **^**(AbN)**	**CTLA-4**^**+ **^**(%)**	**CTLA-4**^**+ **^**(AbN)**
**Good Pathological**					
**Responders**	B	1.39 ± 0.10	249.20 ± 52.00	1.37 ± 0.45	12.67 ± 4.50
**[Group I and II] (n = 9)**	CC	0.50 ± 0.10	150.00 ± 25.00	0.79 ± 0.20	4.75 ± 1.00
	CCvB	p = 0.033*	NS	NS	p = 0.020*
**Poor Pathological**					
**Responders**	B	2.17 ± 1.85	170.29 ± 0.10	1.27 ± 0.30	17.4 ± 2.75
**[Group III and V] (n = 7)**	CC	1.24 ± 0.30	163.00 ± 50.00	1.10 ± 0.24	9.44 ± 2.00
	CCvB	NS	NS	NS	p = 0.010*
**Complete Pathological**					
**Responders [cPR] (n = 6)**	cPR	0.45 ± 0.10	150.00 ± 10.00	0.65 ± 15.00	9.75 ± 0.55
**Healthy Female Donors (n = 10)**	HFDs	0.24 ± 0.02	35.71 ± 2.71	0.21 ± 0.05	4.17 ± 0.75
	cPRvHFDs	p = 0.004**	35.71 ± 2.71	p = 0.015**	p = 0.004**
**Baseline (B) Levels in LLABCs *Versus *(v) Levels Post Surgical Resection (SR) in Different Responders**					
**Study Group Comparisons**		**FOXP3**^**+ **^**(%)**	**CD4**^**+ **^**CD25**^**+ **^**(AbN)**	**CTLA-4**^**+ **^**(%)**	**CTLA-4**^**+ **^**(AbN)**
**Good Pathological Responders**					
**Responders**	B	1.39 ± 0.10	249.20 ± 52.00	1.37 ± 0.45	12.67 ± 4.50
**[Group I and II] (n = 9)**	SR	0.41 ± 0.10	107.33 ± 15.00	0.52 ± 0.10	3.40 ± 1.20
	SRvB	p = 0.022*	p = 0.032*	p = 0.048*	p = 0.010*
**Poor Pathological**					
**Responders**	B	2.17 ± 1.85	170.29 ± 0.10	1.27 ± 0.30	17.4 ± 2.75
**[Group III and V] n = 7)**	SR	0.60 ± 0.15	127.00 ± 12.05	0.75 ± 0.20	4.00 ± 0.50
	SRvB	NS	NS	NS	p = 0.004*
**Complete Pathological**					
**Responders [cPR] Healthy Female** **Donors (n = 10)**	cPR	0.38 ± 0.20	105.22 ±12.00	0.50 ± 0.10	9.75 ± 0.55
	HFDs	0.24 ± 0.02	35.71 ± 2.71	0.21 ± 0.05	3.20 ± 1.10
	cPRvHFDs	p = 0.012**	p = 0.003**	p = 0.024**	p = 0.005**

Values (%, AbN) shown for patients are Means ± SDs. *Statistically significant p values. (student independent *t*-test). NS: Non significant. AbN: Absolute number (cells/μl). Patients studied, n = 16; Good, n = 9; Poor, n = 7; Healthy female donors (HFDs), n = 10. Patients grouped according to pathological responses documented on resected breast specimens.

**Levels significantly greater than documented in HFDs.

After 8 cycles of NAC there was a non significant fall in the AbN of CD4^+^ CD25^+^ cells, compared with pre-treatment levels, in both the good and poor pathological responders. Even in patients with tumours showing cPRs, the AbN CD4^+^ CD25^+^ Tregs were still 4 fold elevated, compared with HFDs (p = 0.008) (Table 2). The lack of the Treg FOXP3 marker in the assay may partly account for these findings.

Eight cycles of NAC had no significant effect on the % of CD4^+^ CD25^+^ CD152^+^ Tregs compared with pre-treatment levels, in either the good or poor pathological responders. Even in patients with tumours showing cPRs, the % of CTLA-4^+^ Tregs were 3 fold elevated, compared with HFDs (p = 0.015) (Table 2).

The AbN of CD4^+^ CD25^+^ CD152^+^ Tregs in the patients with good and poor pathological tumour responses to 8 cycles of NAC showed significant reductions in circulating levels after chemotherapy, compared with pre-treatment levels, (p = 0.020, p = 0.010, respectively). However, even where the tumour showed a cPR there was still a significant elevation of CTLA-4^+^ AbNs, when compared with HFDs (p = 0.004) (Table 2).

Thus, NAC did not abolish the abnormally elevated FOXP3^+^ and CTLA-4^+^ Tregs (%, AbN) in women with LLABCs, albeit there was a variable reduction after 8 cycles of chemotherapy.

### Effect of surgery on blood levels of Tregs (FOXP3^+^, CTLA-4^+^) (n = 16)

Post-surgery, the significant fall in the % of CD4^+^ CD25^+^ CD127^-^ FOXP3^+^ Tregs, documented following NAC, persisted in the good pathological responders (Groups I and II) (p = 0.022). In the poor pathological responders (Groups III and IV) there was a non significant fall in % Tregs. However, even in the cPR patients, % Tregs following surgery was still higher, compared with HFDs (p = 0.012) (Table 2).

Post-surgery, there was a further fall in the AbN of CD4^+^ CD25^+^ cells, when compared with pre-treatment baseline levels, but this was significant only in the good pathological responsders . However, after NAC and subsequent surgery, even in those patients whose tumours showed a cPR, the AbNs were still 3 fold elevated, compared with HFDs (p = 0.003) (Table 2).

Post-surgery, the % of CD4^+^ CD25^+^ CD152^+^ Tregs was reduced further and was now significant (p = 0.048), when compared with pre-treatment baseline levels, but only in the good pathological responders. However, the CTLA-4^+^ Tregs were still abnormally elevated, even in those whose tumours showed a cPR, when compared with HFDs (p = 0.024) (Table 2).

Post-surgery, the AbN of blood CD4^+^ CD25^+^ CD152^+^ Tregs was reduced further, compared with pre-treatment baseline levels, in both the good (p = 0.010) and poor (p = 0.004) pathological responders. However, following completion of treatment, the reduced AbNs of CTLA-4^+^ Tregs in patients whose tumours showed a cPR were still 3 fold elevated, when compared with HFDs (p = 0.005) (Table 2).

Thus, surgical resection of the breast and axilla (potentially curative procedure) did reduce further, to a variable degree and especially in women whose tumours underwent a good pathological response, the residual high levels of Tregs following NAC. However, the blood levels of Tregs following NAC and surgery were still significantly elevated, when compared with HFDs.

### NAC and surgery on blood levels of monocytic (CD11b^+^ CD14^+^ HLADR^-^ CD124^+^ CD33^+^) and granulocytic (CD11b^+^, CD14^-^, CD66b^+^, CD124^+^, CD15^+^) MDSCs (n = 19)

Monocytic MDSC AbNs, were significantly reduced after 4 cycles of NAC, compared with pre-treatment baseline levels, in both good (p = 0.020) and poor clinical responders (p = 0.003) (assessed by MRM after two cycles of AC). This reduction was more pronounced after 8 cycles of chemotherapy in both the good (p = 0.003) and poor clinical responders (p = 0.001). In the good clinical responders, the levels were comparable to those documented in HFDs. In the poor clinical responders the circulating AbNs were below the normal levels documented in HFDs (p = 0.041) (Table 3). Post-surgery, the low levels of blood AbNs of monocytic MDSCs, persisted but were not further reduced in either the good or poor clinical responders.

**Table 3:** Monocytic and Granulocytic MDSCs in Women with LLABCs undergoing NAC and subsequent surgery

**Baseline (B) Levels in LLABCs *Versus *(v) Levels Following Chemotherapy (CC) in Different Responders**				
**Study Group Comparisons**	**Monocytic MDSCs (AbN)**		**Granulocytic MDSCs (AbN)**	
	Good** Clinical Responders (n = 12)	Poor** Clinical Responders (n = 7)	**Good Clinical Responders (n = 12)**	**Poor Clinical Responders (n = 7)**
Baseline (B)	37.00 ± 10.00	25.57 ± 8.00	171.75 ± 25.00	198.67 ± 40.00
CC (4 cycles)	17.25 ± 2.10	7.20 ± 2.10	126.06 ± 15.00	122.50 ± 50.00
CC (4 cycles) v B	p = 0.020*	p = 0.003*	p = 0.032*	NS (p = 0.075)
Baseline (B)	37.00 ± 10.00	25.57 ± 8.00	171.75 ± 25.00	198.67 ± 40.00
CC (8 cycles)	9.45 ± 0.50	4.00 ± 1.20	69.00 ± 20.00	89.90 ± 28.00
CC (8 cycles) v B	p = 0.003*	p = 0.001*	p = 0.006*	p = 0.023*
HFDs	10.20 ± 2.80	10.20 ± 2.80	129 ± 21.00	129 ± 21.00
Post CC (8 cycles) v HFDs	NS	p = 0.041***	p = 0.042***	p = 0.048***
**Baseline (B) Levels in LLABCs *Versus *(v) Levels Post Surgical Resection (SR) in Different Responders**				
	**Good Clinical Responders (n = 12)**	**Poor Clinical Responders (n = 7)**	**Good Clinical Responders (n = 12)**	**Poor Clinical Responders (n = 7)**
Baseline (B)	37.00 ± 10.00	25.57 ± 8.00	171.75 ± 25.00	198.67 ± 40.00
SR	7.11 ± 1.50	3.90 ± 1.50	75.67 ± 9.00	70.00 ± 20.00
SRvB	p = 0.002*	p = 0.046*	p = 0.001*	p = 0.013*
HFDs	10.20 ± 2.80	10.20 ± 2.80	129 ± 21.00	129 ± 21.00
SR v HFDs	NS	p = 0.044***	p = 0.048***	p = 0.044***

Values shown are for baseline, following chemotherapy (CC) and surgery (SR) – Means ± SDs.

* Statistically significant p values (student independent *t*-test); NS: Non significant; ** Good responders : complete or partial response assessed by MRM after two cycles of AC; Poor responders: did not demonstrate a response by MRM after two cycles of AC; *** Levels of AbNs (Monocytic MDSCs, Granulocytic MDSCs) less than that documented in HFDs.

**Table 4:** **Cytokine profiles generated *in vitro *by T Cells from patients with LLABCs undergoing NAC and surgery**

Th1*(pg/ml)	**Responders to NAC**	**LLABCs: Baseline Levels (n = 30)**	**HFDs (n = 8)**	Th2** (pg/ml)	**Responders to NAC**	**LLABCs: Baseline Levels (n = 30)**	**HFDs (n = 8)**
**IL-1β**	GR ***	32.50 ± 10.00 (8 fold reduction) (P = 0.002)	260.00 ± 110.00	**IL-4**	GR	195.64 ± 5.68 (12 fold increase) (P = 0.001)	16.00 ± 9.00
	PR ***	3.33 ± 0.78 (78 fold reduction)(P = 0.001)			PR	197.50 ± 7.50 (12 fold increase) (P = 0.001)	
**IL-2**	GR	702.57 ± 240.00 (2 fold reduction) (P = 0.004)	1400.00 ± 400.00	**IL-5**	GR	156.12 ± 19.89 (6 fold increase) (P = 0.004)	26.93 ± 8.00
	PR	293.33 ± 29.63 (5 fold reduction) (P = 0.001)			PR	602.24 ± 78.58 (24 fold increase)(P = 0.004)	
		reduction) (P = 0.001)					
**INF-γ**	GR	144.76 ± 16.76 (12 fold reduction) (P = 0.009)	1874.89 ± 156.15	**IL-10**	GR	34.42 ± 36.84 (1.7 fold increase) (NS)	20.00 ± 8.00
	PR	78.54 ± 21.42 (24 fold reduction) (P = 0.002)			PR	30.00 ± 6.52 (1.5 fold increase) (NS)	
**TNF-α**	GR	168.78 ± 12.36 (1.3 fold reduction) (P = 0.006)	224.30 ±35.00	Th17*** (pg/ml)	**Responders to NAC**	**LLABCs: Baseline Levels (n = 30)**	**HFDs (n = 8)**
	PR	7.71 ± 3.83 (29 fold reduction) (P = 0.001)		**IL-17A FOXP3**^**-**^**)**	GR	1.95 ± 0.25 2.8 fold increase) P = 0.003)	0.70 ± 0.25
					PR	3.80 ± 0.80 5.4 fold increase) (P = 0.001)	
				**IL-17A (FOXP3**^**+**^**)**	GR	0.04 ± 0.02 (3.8 fold increase) (P = 0.023)	0.01 ± 0.01
					PR	0.05 ± 0.01 (5 fold increase) (P = 0.018)	

*Th1: TNF-β showed no significant changes; **Th2: IL-6 showed no significant changes; *** GR: Good clinical responders (n = 20), PR: Poor clinical responders (n = 10); Responses determined by MRM assessment of the breast following the first two cycles of AC, as part of an eight cycle course of NAC – two or four cycles of AC, followed by six or four cycles of T ± Cap, respectively. **** Th IL-17 ± FOXP3^+^ cells were characterised by FACS analysis.

Granulocytic MDSCs were also very sensitive to chemotherapy, after both 4 and 8 cycles of NAC, and in both the good (p = 0.006) and poor clinical responders (p = 0.023). Following 8 cycles of NAC the AbNs in the good (p = 0.042) and poor clinical responders (p = 0.048) were significantly less than the levels found in HFDs (Table 3). Post-surgery, both the good and poor clinical responders had a persistent but not further reduced level in granulocytic AbNs.

Thus, the MDSCs were very sensitive to NAC, which reduced circulating levels to or below those documented in HFDs, but were not influenced by surgical removal of any residual malignant disease.

### Blood FOXP3^+^, CTLA-4^+^ Tregs (%) and pathological response to chemotherapy (n = 16)

There was a significant correlation (Spearman’s Rho p = 0.04, 2-tailed) between the % of FOXP3^+^ Tregs (prior to NAC) and the subsequent pathological grade of response in the breast cancer following 8 cycles of NAC. The highest levels of FOXP3^+^ Tregs (3.5 ± 0.5%), > 15 fold increase compared with HFDs, were documented in patients whose tumours failed to undergo or had a poor pathological response (grades 1 and 2, respectively) to 8 cycles of NAC (spearmans Rho p = 0.004) (Figure 1A). The lowest levels (0.90 ± 0.2%) of FOXP3^+^ Tregs were seen in the blood of patients whose tumours underwent a cPR (grade 5) following 8 cycles of NAC (Figure 1A).

Post-NAC, there was also a significant correlation (Spearman’s Rho p = 0.05, 2-tailed) between the % of FOXP3^+^ Tregs and the pathological response documented in the breast. The % of blood FOXP3^+^ Tregs in women whose cancers showed a poor pathological response (grades 1 and 2) to NAC (1.50 ± 0.15%) remained high (5 fold increase). Women whose tumours showed a good pathological response (grades 4 and 5) had the lowest levels (0.46 ± 0.25%) of Tregs but these still showed a 2 fold increase, compared with HFDs (0.24 ± 0.02%) (Figure 1A, Table 1).

There was no correlation between the % of blood CTLA-4^+^ Tregs (prior to NAC) and the subsequent pathological grade of tumour response to NAC. However, there was a significant correlation (Spearman’s Rho p = 0.01, 2-tailed) between the % of CTLA-4^+^ Tregs (post-NAC) and the pathological grade of response documented in the breast (Figure 1B). The highest level of post-NAC circulating CTLA-4^+^ Tregs (1.69 ± 0.25%) was seen in patients whose tumours had a poor pathological response. The lowest post-NAC level (0.60 ± 0.05%) was seen in patients whose tumours showed a cPR. However, this was still 2.5 fold higher than the % CTLA-4 Tregs documented in HFDs (Figure 1B, Table 1).

### RT-PCR (FOXP3, CTLA-4) Analysis (n = 16)

**Figure 2:**
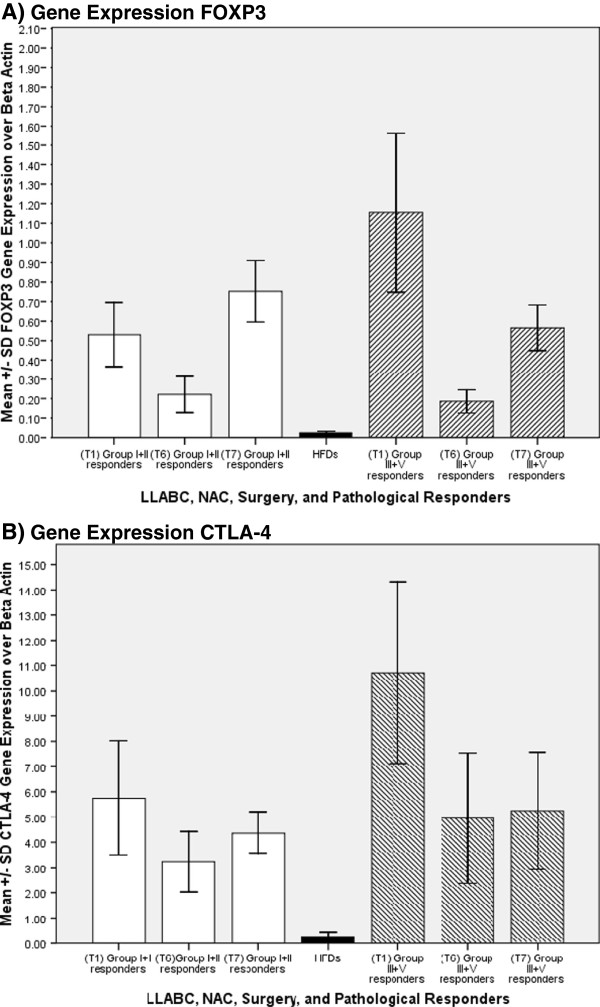
**Gene expression in LLABCs and HFDs**, **(A) FOXP3, (B) CTLA-4: Mean FOXP3 and CTLA-4 gene expression over β actin in the circulation in women with LLABCs (n = 16); baseline (T1), following eight cycles of NAC (T6) and post-surgery (T7).** Patients allocated into groups according to histological responses elicited in the breast cancers after NAC: Group I-cPR; Group II-VGR; Group III-partial response; IV-poor or no response. Statistical analysis: **(A)** FOXP3 (Group I & II): T1 v HFD (p = 0.006); T1 v T6 (NS); T1 v T7 (NS); T6 v HFD (NS); T7 v HFD (p = 0.002). **(A)** FOXP3 (Group III & V): T1 v HFD (p = 0.050); T1 v T6 (NS); T1 v T7 (NS); T6 v HFD (NS); T7 v HFD (p = 0.001). **(B)** CTLA-4 (Group I & II): T1 v HFD (p = 0.011); T1vT6 (NS); T1vT7 (NS); T6 v HFD (NS); T7 v HFD (p = 0.002). **(B)** CTLA-4 (Group III & IV): T1 v HFD (p = 0.038); T1 v T6 (NS); T1 v T7 (NS); T6 v HFD (NS); T7 v HFD (p = 0.041). Group I & II (T1) v III & IV (T1) (NS). Clear columns are good pathological responders whilst shaded columns are poor pathological responders to NAC.

Treg mRNA (FOXP3, CTLA-4) was significantly elevated in both the good and poor pathological responders, compared with HFDs, prior to commencing treatment (T1) (Figure 2A, B). Following NAC (T6) and surgery (T7), there was a non significant fall in FOXP3 and CTLA-4 mRNA levels in all patients. These levels were significantly higher than the levels documented in HFDs: (p ≤ 0.041) (Figure 2A, B). There the substanstial blood transcripts levels paralleled the increase in % Tregs. There was a tendency for poor responders (high Treg levels) to have higher levels of mRNA. However, this did not reach statistical significance and may be a reflection of the relatively small numbers (n = 16) used for the mRNA analysis.

### *In vitro* cytokines produced by T Cells from women with LLABCs, undergoing NAC and surgery (n = 30)

Production of IL-1β was substantially reduced in women with LLABCs; 8 fold in good (p = 0.002) and 78 fold in poor clinical responders (p = 0.001) (tumour responses assessed by MRM after 2 cycles of AC). Following completion of NAC there was a small, non significant increase of IL-1β. Post-surgery, IL-1β levels fell back to pre-treatment levels (data not shown).

IL-2 production was significantly reduced; 2 fold in good clinical responders (p = 0.004) and 5 fold in poor clinical responders (p = 0.001).

Production of INF*-*γ was substantially reduced in women with LLABCs; 12 fold in good clinical responders (p = 0.009) and 24 fold in poor clinical responders (p = 0.002).

Production of TNF-α was reduced in good clinical responders (p = 0.006). Production of TNF-α in poor clinical responders was substantially (29 fold) reduced (p = 0.001).

Thus, there was a more pronounced reduction in the Th1 profile in patients who were poor clinical responders and resistant to NAC. Neither NAC nor surgery altered production of IL-2, INF-γ or TNF-α (data not shown).

Production of IL-4 was substantially increased (12 fold) in women with LLABCs who showed either a good (p = 0.001) or poor clinical response (p = 0.001). Following 8 cycles of NAC there was a reduction of IL-4 production, with further reduction post-surgery; the levels were comparable with HFD levels (data not shown).

Production of IL-5 was substantially increased with good (6 fold) and with poor (24 fold) clinical responders to NAC.

Production of IL-10 was increased (1.7 fold, good clinical responders and 1.5 fold, poor clinical responders), but this was less pronounced than that documented for IL-4 and IL-5 and was non significant.

Production of IL-6 showed no significant changes (data not shown).

Neither NAC nor surgery altered production of IL-5, IL-6 or IL-10 (data not shown).

Intranuclear IL-17A was elevated in Th17 cells, in both good (2.8 fold) (p = 0.003) and poor (5.4 fold) (p = 0.001) clinical responders. IL-17A production by Th17FOXP3^+^ cells was increased 3.8 fold in the good (p = 0.023) and 5 fold in the poor (p = 0.018) clinical responders. NAC and surgery did not alter expression of IL-17A in Th17 ± FOXP3^+^ cells, in either the good or poor clinical responders. The most pronounced increase in IL-17A was seen in the poor clinical responders.

### Representative phenotypic profiles

**Figure 3:**
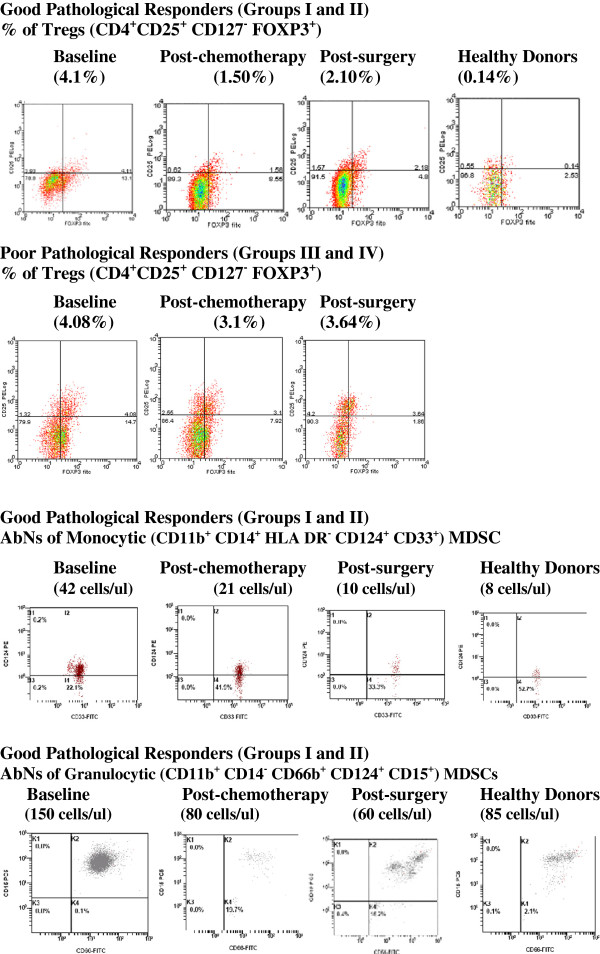
**Representative phenotypic profiles of % CD4**^**+ **^**CD25**^**+**^**CD127**^**- **^**FOXP3**^**+ **^**Tregs in good pathological responders (Groups I and II), 200,000 events acquired, and poor pathological responders (Groups III and IV), 200,000 events acquired.** AbNs of monocytic (CD11b^+^ CD14^+^ HLA DR^-^ CD124^+^ CD33^+^) MDSCs, in good pathological responders (Groups I and 11) 400,000 events acquired, and AbNs of granulocytic (CD11b^+^ CD14^-^ CD66b^+^ CD124^+^ CD15^+^) MDSCs in good pathological responders (Groups I and II), 150,000 events acquired. Data shown are for women with LLABCs, (baseline, post-chemotherapy, post-surgery) and healthy donors.

Figure 3 demonstrates representative phenotypic profiles of FOXP3^+^ Tregs from both good and poor pathological responders to NAC. It also demonstrates representative phenotypic profiles of monocytic and granulocytic MDSCs from good pathological responders to NAC. The profiles illustrate the data shown in Tables 2 and 3, highlighting the abnormally high levels documented in the blood of women with LLABCs, the reductions following NAC and subsequent surgery, and the persistently elevated Tregs but not MDSCs, following completion of treatment.

## Discussion

We have demonstrated that women with LLABCs have significantly and substantially elevated circulating levels (%, AbNs) of Tregs (FOXP3^+^, CTLA-4^+^) and concurrent increased expression of Treg (FOXP3, CTLA-4) mRNA in T cells from blood. Also, circulating AbNs of MDSCs (monocytic, granulocytic) are significantly increased. This data comprehensively expands and defines more precisely the findings of the few studies published, to date, in early breast cancer [11,35-38].

Recent publications have demonstrated elevated blood Tregs in aggressive primary breast tumours, where spread to regional nodes had occurred, and with metastatic disease, albeit Treg specific markers were not always used [38-41]. Not dissimilar findings have been documented in other tumour types [12-14,16]. High circulating levels of MDSCs have also been documented with advanced disease [35].

NAC is being used to treat women with LLABCs [31,32,42]. Some of the beneficial effects are being attributed to modulation of anticancer defences, especially regulatory cells [23-25]. However, the impact of NAC on immunoregulatory cells in women with LLABCs is poorly defined. Aruga et al. (2009) demonstrated a correlation of FOXP3^+^ cells in tumours and subsequent response to NAC. They showed that low numbers of tumour infiltrating FOXP3^+^ cells was associated with a good pathological response but did not characterise blood Tregs [36]. Ladoire et al. (2008) showed that a cPR in breast cancers with NAC was associated with the disappearance of tumour infiltrating FOXP3^+^ Tregs [43]. Diaz-Montero et al. (2009) showed increased circulating MDSCs in patients with breast cancer and correlation with clinical stage, but documented enhanced MDSCs with NAC (ACT) [35].

Our study, to the best of our knowledge, has shown for the first time that the level (%) of circulating FOXP3^+^ Tregs predicted the likely response to NAC – high levels resulting in poor pathological responses in the cancers in the breast. By contrast, low levels of FOXP3^+^ Tregs were associated with a cPR, a recognised surrogate marker of good long-term outcome [31,32,42]. NAC (ACT, ACTCap) significantly reduced Tregs (% FOXP3^+^, AbN CTLA-4^+^), but only after 8 cycles and following a complete or substantial reduction in tumour mass. However, NAC and surgery failed to restore Tregs to the levels found in HFDs. Recently, Oda et al. (2012) described FOXP3^+^ infiltraes in breast cancer as independent predictors of pCR to NAC. The absence of FOXP3^+^ and CD8^+^ infiltrates post NAC was associated with the highest pCR rate (Oda, et al., 2012) [44]. These observations are in keeping with our data and suggest that NAC-induced Treg depletion in the circulation is mirroed within the tumour microenvironment and is crucial for tumour cell death.

Tumour infiltration of breast cancers by Tregs is associated with long-term risk of tumour recurrence and reduced disease-free survival [11,45-47]. However, a recent publication, asscessing FOXP3 expression in triple negative breast cancers, documented improved survival in patients with high levels of tumour infiltrating CD4^+^CD25^+^FOXP3^+^Tregs. The finding, which is at variance with published data, may reflect a unique and, as yet, poorly understood immune interaction in this uncommon pathological subset of breast cancer [48].

We intend to immunohistochemically characterise the Treg profile in the tumour microenvironment of the patients investigated in our study and establish its relevance to subsequent response to NAC.

mRNA levels in the Tregs mirrored the abnormal levels of blood Tregs in women with LLABCs. NAC and surgery non significantly reduced circulating FOXP3^+^ and CTLA-4^+^ Treg mRNA. Post-treatment Tregs mRNAs however were abnormally elevated, compared with levels documented in HFDs. A recent study has documented enhanced FOXP3 and CTLA-4 transcripts in BMCs in breast cancer, but did not assess effect of NAC or surgery [37].

Our study also documented that NAC reduced circulating levels of MDSCs (monocytic, granulocytic), after only 4 cycles. This was more pronounced after 8 cycles of NAC and the MDSCs were not reduced further by surgery, suggesting a direct cytotoxic effect on MDSCs, irrespective of tumour burden. These findings are at variance with the enhancement documented with ACT in a recent study [35]. The different time course used may be a factor. In a study in patients with advanced non-small cell lung cancer granulocytic (CD11b^+^ CD14^-^ CD15^+^) MDSCs were increased in blood. Following chemotherapy and/or surgery, the level of these MDSCs fell significantly supporting the findings of NAC in breast cancer in our study [49].

Different subsets of MDSCs in humans are able to induce both Th17 effector cells and immunoinhibitory Tregs when co-cultured with CD4^+^ T cells [50]. MDSCs can also induce differentiation of FOXP3^+^ Tregs from monocyte-derived Th17 cells [50]. Our study suggests that the beneficial effects of NAC may be due, in part, to a reduction of immunoregulatory cells by a direct effect on Tregs and/or through inhibition of generation by MDSCs. The presence of elevated residual Tregs, albeit low, may reflect persistent occult micrometastases and need for further therapy, designed to abolish the remaining foci of regulatory cells [10,29,33,34]. Further careful studies need to be done to address this issue and ascertain possible beneficial clinical outcome from such an approach.

The Th1 profile (IL-1β, IL-2, INF-γ, TNF-α) from women with LLABCs was substantially and significantly reduced, compared with HFDs. The most pronounced reduction occurred in patients whose tumours responded poorly to NAC. By contrast, the Th2 profile (IL-4, IL-5) was substantially and significantly increased; IL-10 was non significantly increased, and IL-6 showed no changes. NAC with or without surgery had no effect on Th1 and Th2 profiles, except for IL-4, which resembled levels found in HFDs following chemotherapy. Polarisation of cytokine responses has been described in breast cancer [51]. Our study characterised this more comprehensively, documenting the inability of NAC and surgery to alter the majority of these abnormal changes, even in the presence of significant reduction of tumour burden and levels of regulatory cells. This complex and ongoing immune suppression suggests a chronic or permanent dysfunction of host defences induced by the malignant state. Careful and long-term monitoring of this dysfunctional immune state may provide insight as to likely long-term clinical outcome.

Th17 cells play a crucial role in inflammation and autoimmunity, but their function in cancer is unclear [52,53]. Animal studies have shown a close association between IL-17 production by tumour infiltrating lymphocytes and destruction of tumour cells by induction of Th1 lymphocytes and CTL antitumour responses [54,55]. TGF-β and IL-6 induce Th17 differentiation, amplified by IL-1β and TNF-α. Increased Th17 cells have been documented in blood, lymph nodes and human tumours [16,52]. Data suggests they may enhance anticancer defences in man [53,56]. A recent study in NODscid mice bearing human ovarian cancer and transfer of autologous Th17 human cells indicated an anticancer collaboration between Th17 cells and CD8^+^ CTLs [53]. High levels of tumour infiltrating Th17 cells in breast cancer has been shown to be associated with improved prognosis and reduction of metastases [56]. In our study, production of IL-17A by Th17 lymphocytes (±FOXP3^+^) was significantly increased in good (2.8 fold) and poor (5 fold) clinical responders. NAC had no demonstrable effect on IL-17A production. High dose cyclophosphamide in animals and metronomic doses in humans can induce the differentiation of Th17 cells [57]. The impact of high dose cyclophosphamide (as in our NAC protocol) in man has not been studied, but appears to lack any overt regulatory effect.

## Conclusions

This is the most comprehensive and definitive documentation in women with LLABCs, demonstrating abnormal circulatory levels (numbers, gene expression) of regulatory cells, Th1/Th2 cytokine polarization, refractory to treatment, and a concomitant enhanced Th17 response. This complex interplay is associated with chemoresistance predicting the likely response to NAC in women with LLABCs and is a possible biological marker of occult disease. Our findings infer a chronic and/or possibly permanent dysfunction of crucial host defences, even after apparently curative treatment. NAC and surgery are important standard treatments for women with LLABCs and other cancers. However, novel immunotherapeutic strategies (monitoring and targeting treatment to inhibit or delete regulatory cells) need to be designed and evaluated to address the important results documented in our study.

## Abbreviations

A: Adriamycin; AbN: Absolute Number; B: Baseline; C: Cyclophosphamide; Cap: Capecitabine; CC: Completion of Chemotherapy; CD: Cluster of Differentiation; cPR: Complete Pathological Response; CTLA-4: Cytotoxic T Lymphocyte Antigen 4; DC: Dendritic Cell; FACS: Fluorescent Activated Cell Sorter; FOXP3+: Forkhead Box P3; FRAME: Funds for the Replacement of Animals in Medical Experiments; GR: Good Clinical Responder; HFD: Health Female Donor; IL: Interleukin; INF-γ: Interferon Gamma; LLABC: Large Locally Advanced Breast Cancer; MAbs: Monoclonal Antibodies; MDSC: Myeloid-derived Suppressor Cells; MO: Metastatic Disease (No Evidence of Disease); MRM: Magnetic Resonance Mamography; mRNA: Messenger RNA; NAC: Neoadjuvant Chemotherpy; NK/NKT: Natural Killer/Natural Killer T (Cell); NO-2: Nodal Status (Clinical); PCR: Polymerase Chain Reaction; PR: Poor Clinical Responder; RT: Reverse Transcriptase; SR: Surgical Resection; T: Doxecetaxel; T2-4: Tumour Size (Clinical); TCM: Tissue Culture Medium; TGF-β: Transforming Growth Factor Beta; Th: T Helper (Cell); TNF-α: Tumour Necrosis Factor Alpha; Treg: T Regulatory Cell

## Competing interests

The clinical trial, from whose patients blood samples were collected for the study, was supported by educational grants from Sanofi-Aventis UK, Roche UK and Chughai UK.

## Authors’ contributions

Literature Search: CV, JE, GC, MS, JJ, OE. Figures: CV, OE. Study design: CV, JE, OE. Data collection: CV, JE, AR, AB, GC, OE. Data interpretation: CV, JE, AB, GC, OE. Laboratory assays: CV, AR, AB. Writing of manuscript: CV, JE, OE. Review of manuscript: CV, JE, AR, AB, GC, MS, JJ, OE. All authors read and approved the final manuscript.
